# Screening and identification of grain sorghum germplasm for salt tolerance at seedling stage

**DOI:** 10.3389/fpls.2025.1610685

**Published:** 2025-06-20

**Authors:** Yu Wang, Dongyang Li, Chunjuan Liu, Xiaolong Shi, Yan Huang, Chang Liu, Yufei Zhou

**Affiliations:** ^1^ College of Agronomy, Shenyang Agricultural University, Shenyang, China; ^2^ Baicheng Academy of Agricultural Sciences, Baicheng, China

**Keywords:** sorghum, salt tolerance evaluation, principal component analysis, cluster analysis, biologiclal evaluation

## Abstract

**Introduction:**

Sorghum is characterized by its salt tolerance, and holds great potential for cultivation in saline-alkali soils.

**Methods:**

This study aimed to comprehensively evaluate the salt tolerance of grain sorghum germplasm. The experiment was conducted with 188 grain sorghum germplasm accessions selected to investigate the morphological and physiological index of seedlings under 150 mM NaCl stress. A comprehensive salt tolerance evaluation system was constructed using six indicators: shoot length (SL), root length (RL), shoot fresh weight (SFW), root fresh weight (RFW), shoot dry weight (SDW), and root dry weight (RDW). The salt tolerance level of the 188 accessions was evaluated using methods including the salt tolerant index (SI), the Spearman correlation analysis, the membership function analysis, the principal component analysis (PCA), and the cluster analysis.

**Results:**

The results were classified into five categories: highly salt tolerant, salt-tolerant, moderate, salt-sensitive, and highly salt-sensitive. The comprehensive evaluation revealed significant variability in salt tolerance among the sorghum germplasm, with an overall trend of normal distribution. The loadings of shoot growth parameters (SFW and SDW) were relatively high, explaining most of the information in the first principal component. Therefore, shoot growth status can be used as an important standard for evaluating salt tolerance in sorghum. Among the accessions, LCS177 and LCS234 exhibited extremely high salt tolerance, while LCS140 and LCS181 showed highly sensitive of salt tolerance. Further analysis of the physiological characteristics of salt tolerance in the selected extreme accessions revealed that under salt stress, the increases in proline, soluble protein, and soluble sugar contents were significantly higher in the salt-tolerant accessions LCS177 and LCS234 than in the salt-sensitive accessions LCS140 and LCS181. In terms of oxidative stress, the activities of SOD, POD, and CAT were significantly higher in the salt-tolerant accessions LCS177 and LCS234, while the content of MDA was significantly lower in salt-tolerant accessions compared to that of in salt-sensitive accessions LCS140 and LCS181.

**Discussion:**

The results of this study provide a material basis for the improvement of salt tolerance in sorghum germplasm resources and for the breeding of salt-tolerant sorghum varieties.

## Introduction

Sorghum is one of the important food crops with good salt tolerance ability and has a high potential for saline soil planting. Accumulation of salt ions is a major factor limiting plant growth and crop yield (Yu et al., 2021). Salinization leads to significant loss of soil nutrients and destroys the healthy structure of the soil, resulting in lower yields and quality crops (Li et al., 2021). According to statistics, the current area of saline soil in the world is about 831 million ha, equivalent to 10% of the global land area (Zhao et al., 2022). Breeding salt-tolerant crops that can be grown in saline soil is one of the key strategies to proactively address this challenge (Melino and Tester, 2023).

Selection and breeding of salt-tolerant crops can not only effectively improve the adaptability of crops to salt stress but also achieve stable and high yields under salt soil conditions (Qin et al., 2020). The prerequisite for selecting and breeding salt-tolerant crops is the identifying and screening of basic germplasm materials for salt tolerance, and good salt-tolerant basic materials are the key to selecting and breeding excellent salt-tolerant varieties (Shelake et al., 2022; Singh et al., 2021). Tian et al. (2024) employed a variety of analytical methods to evaluate the salt tolerance of maize, identifying the appropriate NaCl concentration for screening salt-tolerant maize varieties. They utilized membership function analysis and principal component analysis (PCA) to determine six principal components, including plant height, peroxidase (POD) activity, and proline content, which were used to screen salt-tolerant maize varieties. Li et al. (2020) evaluated 552 sunflower germplasm and found that the germination index (GI) and germination energy index (GEI) were the most highly correlated characters with salt tolerance, and a method for evaluating the salt tolerance of sunflower seed germination was established, which provides a basis for selecting and breeding salt-tolerant varieties. Zhang et al. (2021) assessed salt tolerance of 114 rice varieties at gemination stage with different salt concentration treatments. The principal component analysis, membership function analysis, and correlation analysis were used in the evaluation and the salt tolerant indexes for salt stress evaluation were identified. Gholizadeh et al. (2022) assessed 110 Iranian wheat germplasm materials for salt stress tolerance using salt tolerance membership function values (MFVS). Ten highly tolerant genotypes were screened, and five morphological and agronomic traits were identified as indicators for screening salt stress tolerant wheat.

Salinity is one of the major adversity stresses affecting sorghum growth and yield. Several studies have been focused on screening salt-tolerant sorghum germplasm for salt tolerance. Ding et al. (2018) utilized MFVS as a comprehensive tool for assessing the salt-tolerant performance of genotypes and comprehensively examined several physiological and agronomic characteristics at different developmental stages. Based on the results of the experiments, the different sweet sorghum germplasm was categorized as salt-tolerant, moderately salt-tolerant, salt-sensitive and highly salt-sensitive. Oberoi et al. (2023) conducted salt tolerance screening of traditional sorghum varieties at seedling stage by measuring morphological and physiological parameters such as stem length, root length, total sugar content, and total chlorophyll content, they identified several superior varieties with high salt tolerance, providing valuable genetic resources for breeding programs. Meanwhile, Sun et al. (2012) screened hybrid sorghum varieties for salt tolerance. These studies highlight the potential of sorghum and hybrid varieties for enhanced salt tolerance, but there is still a gap in the screening of salt tolerant grain sorghum germplasm.

This study focused on 188 core germplasms of grain sorghum, targeting the seedling stage, a critical phase in sorghum growth and development, to conduct salt tolerance identification and screening. The seedling stage is highly sensitive and responsive to salt stress, making it more precise for screening salt-tolerant germplasms compared to other stages such as germination and reproductive growth. Good salt tolerance at this stage is of great significance for the successful establishment and early growth of sorghum in saline-alkali soils. In this study, a comprehensive evaluation and cluster analysis of the germplasm population were carried out using the relative salt tolerance coefficient (SI), membership function, and PCA. Compared with traditional single indicators, these methods can more comprehensively and accurately assess the salt tolerance of sorghum germplasms. Furthermore, the salt tolerance of the extreme germplasms selected was verified from the physiological aspect of salt tolerance. This integrated research approach, from morphological parameters to physiological characteristics, is more in line with practical production needs and provides a reliable reference for salt-tolerant breeding of grain sorghum. Previous studies on sorghum salt tolerance have mainly focused on traditional or hybrid sorghum varieties, while this study focuses on the salt tolerance screening of grain sorghum germplasm populations, which can provide material support for forage and grain sorghum breeding and can be directly used for germplasm improvement and breeding.

## Materials and methods

### Experimental material

A total of 188 germplasm resources of grain sorghum were used in this research. The germplasm resources were from all over the world, 125 were from China (30 from Heilongjiang Province, 30 from Jilin Province, 30 from Liaoning Province, 25 from Shanxi Province and 10 from Guizhou Province) and 63 were from other countries (27 from the US, 19 from India, 5 from Australia and 12 from Africa). The details of them are shown in **Supplementary Table 1**.

### Salt stress treatment

For each variety, sorghum seeds with uniform size and full grains were selected. The seeds were surface sterilized with 10% sodium hypochlorite (NaClO) solution for 3–5 min, then thoroughly rinsed with distilled water. Afterward, they were placed in Petri dishes lined with moist filter paper and incubated in the dark at 25°C. After two days of germination, the seedlings were transferred into hydroponic boxes and grown in a constant-temperature growth chamber. The cultivation conditions were as follows: day/night temperatures of 28°C/25°C, with 12-hour light/12-hour dark cycles, and a light intensity of 280 μmol m^−2^ s^−1^. After three days of cultivation in distilled water, the solution was replaced with 1/2 Hoagland nutrient solution. Following another three days of growth, salt stress treatment was initiated by adding 150 mM NaCl to the nutrient solution (Tang et al., 2024). The nutrient solution without NaCl was used as the control. After 7 days of salt stress treatment (at which point the sorghum seedlings had developed approximately five leaves), seedlings with similar growth status were selected from each treatment group for subsequent measurements (Liu et al., 2023).

### Measurement of seedling phenotypic data under salt tolerance

To evaluate the salt tolerance of 188 sorghum germplasm resources at the seedling stage, shoot length (SL), root length (RL), shoot fresh weight (SFW), root fresh weight (RFW), shoot dry weight (SDW) and root dry weight (RDW) were measured. Shoot length and root length were measured with a steel ruler; shoot fresh weight and root fresh weight were weighed with an electronic balance, shoot dry weight and root dry weight were measured by drying in an oven at 105°C for 2 h, followed by drying at 70°C for 48 h, and finally weighed using an electronic balance. Each parameter was measured in five replicates.

### Data analysis and salt tolerance evaluation

1. The salt tolerance index (SI) was calculated as the ratio of the data derived from the salt stress (S) treatment and the control (CK), which was applied to the same germplasm for each trait using the following equation.


$$SI_{ij}=\frac{X_{ijs}}{X_{ijck}}$$

(Where SI_ij_ is the salt tolerance index for trait (j) of variety (i); X_ijs_ and X_ijck_ are the values of trait (j) of variety (i) assessed under salt stress (S) and control (CK) treatments, respectively.)

2. Membership Value [U(X_i_)].


$$U(X_{i})=\frac{\left(X_{i}-X_{min}\right)}{\left(X_{max}-X_{min}\right)}$$

U(X_i_): the membership Value of the i-th composite indicator; (i=1, 2, 3……, n); X_i_: the measured value of the i-th composite indicator; X_max_: the maximum value of the i-th composite indicator; X_min_: the minimum value of the i-th composite indicator.

3. Weights [W_i_].


$$W_{i}=P_{i}/\sum_{i=1}^{m}P_{i}$$

(W_i_: weight of the i-th composite indicator among all composite indicators; (i=1, 2, 3……, n); P_i_: contribution of the i-th composite indicator).

4. Principal component analysis (PCA).


$$F=\sum_{i=1}^{k(n)}F_{i}f_{i}$$

W_i_ represents the weights, i.e., the variance contribution of each principal component, and f_i_ represents the factor score of the i-th factor.

5. Comprehensive evaluation value [D].


$$D=\sum_{i=1}^{m}[U(X_{i})\times W_{i}]$$

U(X_i_): the membership Value of the i-th composite indicator; W_i_: the weight of the i-th composite indicator among all composite indicators; (i=1, 2, 3……m).

### Determination of physiological parameters in sorghum seedlings

1. Measurement of osmotic stress-related indicators:

Soluble sugar content was determined by anthrone colorimetry (Xu et al., 2022). Determination of soluble protein content by Caulmers Brilliant Blue method (G250) (Guo et al., 2022). The free proline content was calculated by color development in the ninhydrin reaction and its absorbance was measured spectrophotometrically at 515 nm (Abraham et al., 2010).

2. Measurement of antioxidant-related indicators:

The tissue fluid to be measured was first extracted by homogenizing 0.5 g of leaf tissue in pre-cooled 50 mM phosphate buffer (pH=7.8), and then the homogenate was centrifuged at 10,000 × g for 20 min at 4°C. The supernatant was used for the determination of Superoxide Dismutase (SOD), POD and Catalase (CAT).

SOD activity:

1. Reaction System Configuration: A clean cuvette was taken, and the following reagents were added sequentially: 750 mmol of NBT (Nitroblue Tetrazolium), 20 mmol of riboflavin, 130 mmol of methionine, 100 mmol of EDTA, and 50 mM sodium phosphate buffer (pH=7.8). An appropriate volume of enzyme solution was then added to reach the desired total volume (the specific volume should be determined based on experimental requirements). The mixture was gently mixed to ensure that all components were fully dissolved and uniformly mixed. The prepared reaction mixture was placed under a light intensity of 4000 lx for 20 minutes to initiate the SOD catalyzed reaction, which involves the dismutation of superoxide anions by SOD to inhibit the reduction and discoloration of NBT. Simultaneously, a control group with an identical reaction mixture was set up but kept in the dark without light exposure to determine the natural reduction of NBT, thereby allowing for the accurate calculation of the inhibitory effect of SOD.
2. Measurement and Calculation of Results: After irradiation, the reaction mixture was quickly transferred to a cuvette and the absorbance was measured at 560 nm using a spectrophotometer. The SOD activity was calculated based on the measured absorbance values, with the amount of SOD required to inhibit the reduction of nitroblue tetrazolium (NBT) by 50% defined as one unit of enzyme activity. Biological replicates were performed three times to ensure the reliability and accuracy of the data and to minimize the impact of random errors on the results.

POD Activity:

1. Reaction System Configuration: In a sodium phosphate buffer (100 mM, pH=6.0), 0.25 mmol of guaiacol and 3 mM hydrogen peroxide were added, followed by an appropriate volume of enzyme solution to reach the desired total volume (the specific volume was determined experimentally). The mixture was gently shaken to ensure thorough mixing and dissolution of all components, forming the reaction mixture. Under the catalysis of POD enzyme, guaiacol is oxidized by hydrogen peroxide to form a red quinone compound, and the change in absorbance can be used to reflect POD activity.
2. Measurement and Calculation of Results: The reaction mixture was placed in a cuvette and immediately inserted into a spectrophotometer to continuously monitor the change in absorbance at 470 nm. The increase in absorbance per minute was recorded. One unit of POD activity (UPOD) was defined as the amount of enzyme that corresponds to an absorbance increase of 0.1 per minute per gram of fresh weight (g^-1^ FW) at 470 nm.

CAT Activity:

1. Reaction System Configuration: Three milliliters of sodium phosphate buffer (50 mM, pH=7.0) were taken in a cuvette, and 3 mM hydrogen peroxide was added, followed by an appropriate volume of enzyme solution. The mixture was gently mixed to ensure full contact between hydrogen peroxide and CAT enzyme, initiating the reaction for CAT to catalyze the decomposition of hydrogen peroxide into water and oxygen. The decrease in absorbance is related to CAT activity.
2. Measurement and Calculation of Results: The reaction mixture was placed in a cuvette and the absorbance was measured at 240 nm using a spectrophotometer. The decrease in absorbance per minute was recorded. One unit of CAT activity was defined as the amount of enzyme that corresponds to a decrease in absorbance of 0.01 per minute per gram of fresh weight (g^-1^ FW) at 240 nm (Du et al., 2019).
3. Malondialdehyde (MDA) was determined by Thio barbituric acid (TBA) method (Xu et al., 2020).

### Data statistics

The data of each trait measured in the experiment were organized using Microsoft Excel 2019 and SPSS 25 software. The mean, maximum and minimum values as well as the coefficient of variation (COV) (standard deviation/mean) × 100% and salt tolerance index analysis were counted for each sorghum variety and each of its traits. The data collated in Excel were analyzed by SPSS 25 and origin 2022 for correlation analysis, principal component analysis, comprehensive evaluation, and cluster analysis among the traits of each parameter.

## Results

### Distribution of salt tolerance indicators and salt tolerance index in core germplasm populations under salt stress

Six morphological indicators of 188 sorghum materials were statistically analyzed under salt stress conditions and normal conditions (**Supplementary Table 1**). Under normal conditions, the results showed that the mean values of shoot length, root length, shoot fresh weight, root fresh weight, shoot dry weight and root dry weight of different germplasm of sorghum were 27.894 cm, 16.689 cm, 0.760 g, 0.288 g, 0.073 g and 0.022 g, respectively. The CoVs were 17%, 18%, 42%, 37%, 46% and 67%, respectively. Under salt stress, the mean values of shoot length, root length, shoot fresh weight, root fresh weight, shoot dry weight and root dry weight of sorghum were 18.810 cm, 13.321 cm, 0.343 g, 0.143 g, 0.050 g and 0.015 g, respectively. The coefficients of variation were 16%, 22%, 34%, 33%, 41% and 70%, respectively. Salt stress decreased shoot length, root length, shoot fresh weight, root fresh weight, shoot dry weight and root dry weight by 32.57%, 20.18%, 54.87%, 50.35%, 31.51% and 31.82%, respectively, compared to the control (**Table 1**). It is evident that salt stress affected the morphological indexes of seedlings to different degrees, and there were large differences among the varieties.

**Table 1:** Variation of different indicators.

Norm	Control subjects			Salt stress			Relative value		
	Average	COV (%)	Range	Average	COV (%)	Range	Average	COV (%)	Range
SL	27.89	16.92%	18.00-39.60	18.81	16.12%	8.63-27.0	0.69	17.48%	0.43-1.11
RL	16.69	17.63%	9.75-27.50	13.32	21.96%	7.00-21.1	0.81	18.75%	0.31-1.25
SFW	0.76	41.61%	0.19-3.23	0.34	33.57%	0.20-0.42	0.48	32.48%	0.10-1.18
RFW	0.29	36.97%	0.05-0.19	0.14	33.24%	0.10-0.17	0.53	36.70%	0.13-1.61
SDW	0.07	46.38%	0.02-0.35	0.05	41.16%	0.03-0.06	0.73	34.58%	0.21-2.26
RDW	0.02	66.61%	0.00-0.19	0.02	70.71%	0.01-0.02	0.73	45.86%	0.24-3.58

**Figure 1:**
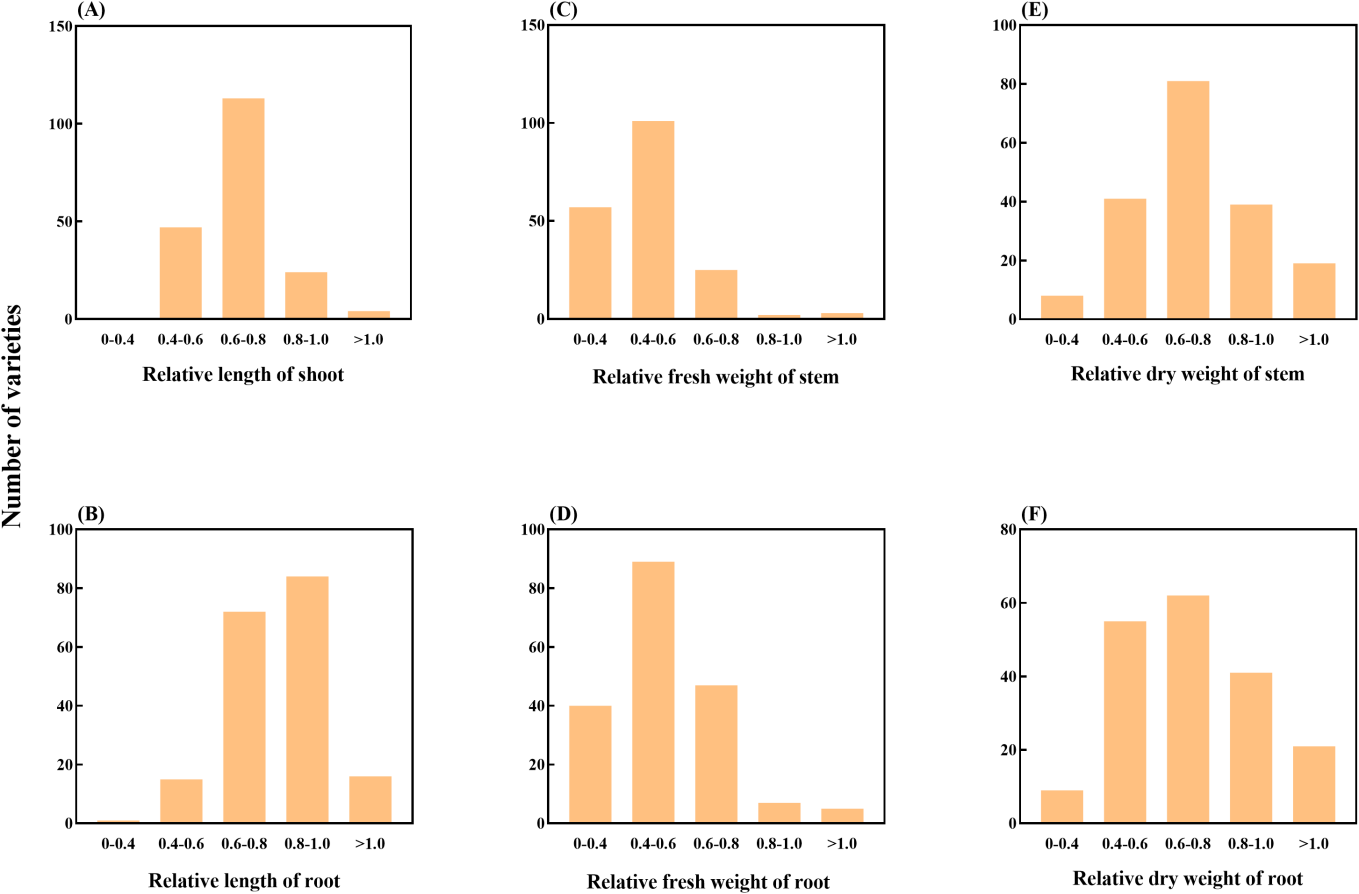
Classification of 188 sorghum accessions according to the relative values of various parameters, **(A)** relative length of stem; **(B)** relative length of root; **(C)** relative fresh weight of stem; **(D)** relative fresh weight of root; **(E)** relative dry weight of stem; **(F)** relative dry weight of root. The x-axis of each figure represents categorical frequency distribution, while the y-axis indicates the number of accessions within each proportion category. The overall annotation is provided on the far left side of the figure for clarity.

The SI of sorghum can well reflect the sensitivity of sorghum to salt stress (**Supplementary Table 2**). By calculating the SI indices for shoot length, root length, shoot fresh weight, root fresh weight, shoot dry weight and root dry weight, it was found that the shoot length of LCS53, ZYF84 and LCS181 germplasm was significantly suppressed under NaCl treatment (*P* < 0.05), but traits of the germplasm of LCS177, ZYF150 and ZYF201 were only slightly affected, showing obvious differences in salt tolerance. After treatment with 150 mM NaCl, 47 germplasms had SI values lower than 0.6 for SL, with most SI values ranging from 0.6 to 1.0 (137 germplasms). The SI value distribution of RL was similar to that of SL, with four germplasms exhibiting SI values greater than 1.0 (**Figures 1A, B**), while the majority of germplasms had SI values above 0.6.

For the SI values of SFW and RFW under 150 mM NaCl treatment, only a few germplasm accessions (5 and 12, respectively) had SI values greater than 0.8. However, a total of 158 and 129 germplasm accessions had SI values below 0.6 for SFW and RFW, respectively (**Figures 1C, D**). A similar trend was observed for the SI values of SDW and RDW. Under 150 mM NaCl treatment, most germplasm accessions (180 and 179, respectively) exhibited SI values between 0.4 and 1.0.

In summary, all the indicators of sorghum showed some degree of changes under salt stress conditions, but the degree of changes varied. Therefore, it is necessary to screen key indicators from them to assess the salt tolerance characteristics of different varieties.

### Correlation analysis between different SIs of each trait of sorghum seedlings

**Figure 2:**
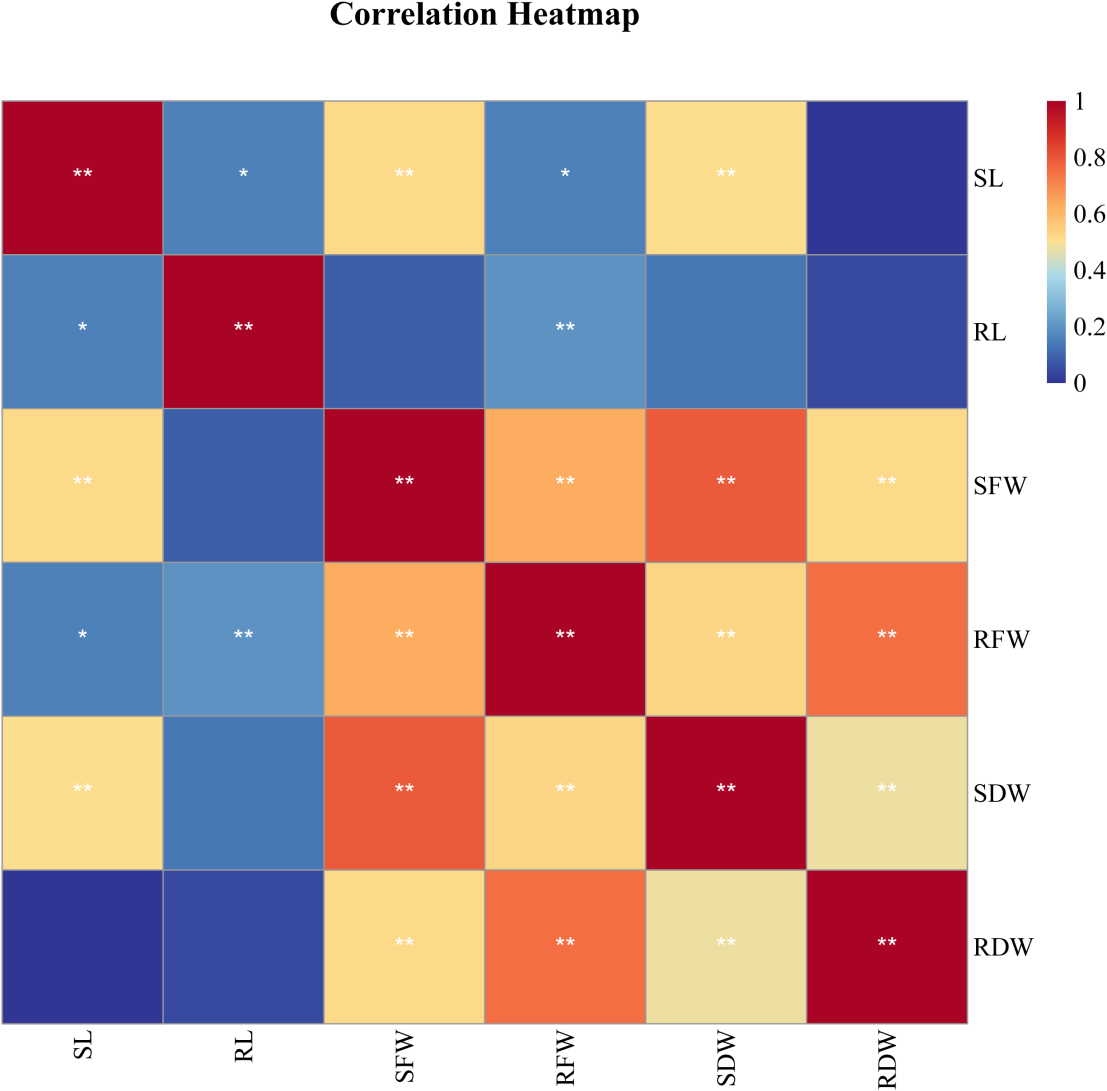
Correlation analysis of salt tolerance index of different traits of 188 sorghum lines character parameters *Significantly relevant at the 0.05 level; **Significantly relevant at the 0.01 level.

Correlation analysis is used to explore the relationships between two or more variables. In this study, correlation analysis based on the salt tolerance coefficient (SI) was conducted for various traits (**Figure 2**). The results showed that the correlations between most traits reached significant or highly significant levels, revealing their interconnections. Under salt stress conditions, SL was significantly positively correlated with SFW and SDW (*P* < 0.05), indicating an association in the response of these traits to salt stress. SFW was highly significantly positively correlated with RFW, SDW, and RDW (P < 0.01), reflecting the close relationship between shoot and root growth. RFW was also highly significantly correlated with SDW and RDW (P < 0.01), and similarly, SDW was highly significantly positively correlated with RDW (P < 0.01). Overall, there were certain differences in the correlations between different SI indicators. Therefore, it is necessary to select representative traits and combine them with multivariate statistical methods for comprehensive evaluation to more accurately identify the salt tolerance of the germplasm.

### Principal component analysis of salt stress tolerance of different sorghum varieties in seedling stage

By analyzing the coefficient of variation and correlation of each indicator, the SI of six morphological traits (SL, RL, SFW, RFW, SDW and RDW) were selected for principal component analysis. Based on the KMO and Bartlett’s sphericity tests (**Table 2**), the KMO value was 0.733, which falls within the recommended range (0.7–0.8) for PCA. Additionally, Bartlett’s test of sphericity yielded a p-value significantly lower than 0.05, indicating that the variables are highly correlated. Consequently, these results confirm that the data are suitable for PCA.

**Table 2:** KMO and Bartlett’s test.

Statistics	KMO value	0.733
Bartlett Sphericity Check	Approximate chi-square	361.743
	df	15
	P-value	0

According to the analysis of the eigenvalues and cumulative contribution rates of the six traits in **Table 3**, the results showed that the six indicators were divided into three principal components, with a cumulative contribution of 81.51%, which satisfied with the principle of principal component extraction. The eigenvalue of principal component 1 was 2.80, with 46.62% variance explained, the eigenvalue of principal component 2 was 1.18, with 19.62% variance explained, and the eigenvalue of principal component 3 was 0.92, with 15.27% variance explained, with a cumulative contribution rate of 81.51% (**Table 3**). Therefore, the six relevant individual indicators can be transformed into three new composite indicators (CIs) for analyzing the salt tolerance characteristics of sorghum varieties and are able to retain the majority of the information from the original indicators.

**Table 3:** Characteristic values and contribution rates of six main agronomic traits of sorghum.

PC	Eigenvalue	VER (%)	CCR (%)
1	2.797	46.620	46.620
2	1.177	19.616	66.236
3	0.916	15.274	81.510

The correlation coefficients of these three principal components with the six morphological indicators can be seen in the factor loading matrix (**Table 4**), which reflects the correlation between them. The first principal component mainly responded to the growth of shoot, and the positive and large loading values were shoot fresh weight, shoot dry weight, and root fresh weight, with loading values of 0.879, 0.838, and 0.806. The positive and large loading values of the second principal component were shoot length and root length, with loading values of 0.651 and 0.595. The positive and large loading value of the third principal component were the root fresh weight, with a loading value of 0.759.

**Table 4:** Principal component matrix of six main trait parameters of sorghum core collection.

Indicator	I	II	III
Shoot fresh weight	0.879	-0.021	-0.186
Shoot dry weight	0.838	0.069	-0.211
Root fresh weight	0.806	-0.207	0.252
Shoot length	0.523	0.651	-0.361
Root dry weight	0.589	-0.592	0.260
Root length	0.228	0.595	0.759

### Comprehensive analysis of salt stress tolerance of different sorghum varieties in seedling stage

Based on the results of the principal component matrix analysis, the original six indicators were converted into three main components based on the score coefficient matrix for each factor (**Supplementary Table 3**), with the following functional expressions for each of the first three principal components:

y_1_ = 0.313x_1_+0.136x_2_+0.526x_3_+0.482x_4_+0.501x_5_+0.352x_6_
y_2_ = 0.600x_1_+0.548x_2_-0.019x_3_-0.191x_4_+0.064x_5_-0.546x_6_
y_3_=-0.377x_1_+0.793x_2_-0.194x_3_+0.263x_4_-0.221x_5_+0.272x_6_

Where x_1_ represents shoot length, x_2_ represents root length, x_3_ represents shoot fresh weight, x_4_ represents root fresh weight, x_5_ represents shoot dry weight, and x_6_ represents root dry weight, and y_1_, y_2_, and y_3_ represent composite index 1 (CI_1_), composite index 2 (CI_2_), and composite index 3 (CI_3_), respectively. The comprehensive evaluation value (D) of each variety was calculated according to Equations 2 and 5. Among them, the higher the composite evaluation value (D), the stronger the salt tolerance of the variety (**Supplementary Table 4**). Among the comprehensive salt tolerance rankings (D values) of 188 sorghum germplasms, the top ten accessions were LCS177 (0.686), 2018-413 (0.530), ZYF150 (0.527), LCS234 (0.492), ZYF325 (0.476), LCS29 (0.459), LCS137 (0.451), ZYF29 (0.447), ZYF201 (0.444), and LCS220 (0.440). LCS177 (0.686), which ranked first, was 0.156 higher than the second-ranked 2018-413 (0.530) and 0.246 higher than the tenth-ranked LCS220 (0.440), demonstrating a significant advantage in salt tolerance. The ten lowest-ranked accessions were LCS53 (0.107), LCS181 (0.117), LCS140 (0.142), ZYF88 (0.152), LCS99 (0.160), ZYF146 (0.160), LCS22 (0.168), LCS165 (0.168), LCS80 (0.173), and LCS151 (0.178). The lowest-ranked LCS53 (0.107) was only 0.010 lower than LCS181 (0.117), which ranked second to last, and 0.071 lower than LCS151 (0.178), which ranked tenth from the bottom. While the overall D values of these low-tolerance accessions were low, the differences among them were relatively small. Overall, the top ten ranked accessions showed greater differences in D values, with LCS177 standing out significantly from the others, whereas the lower-ranked accessions exhibited relatively minor differences in their D values.

### Cluster analysis of salt stress tolerance of different sorghum varieties in seedling stage

**Figure 3:**
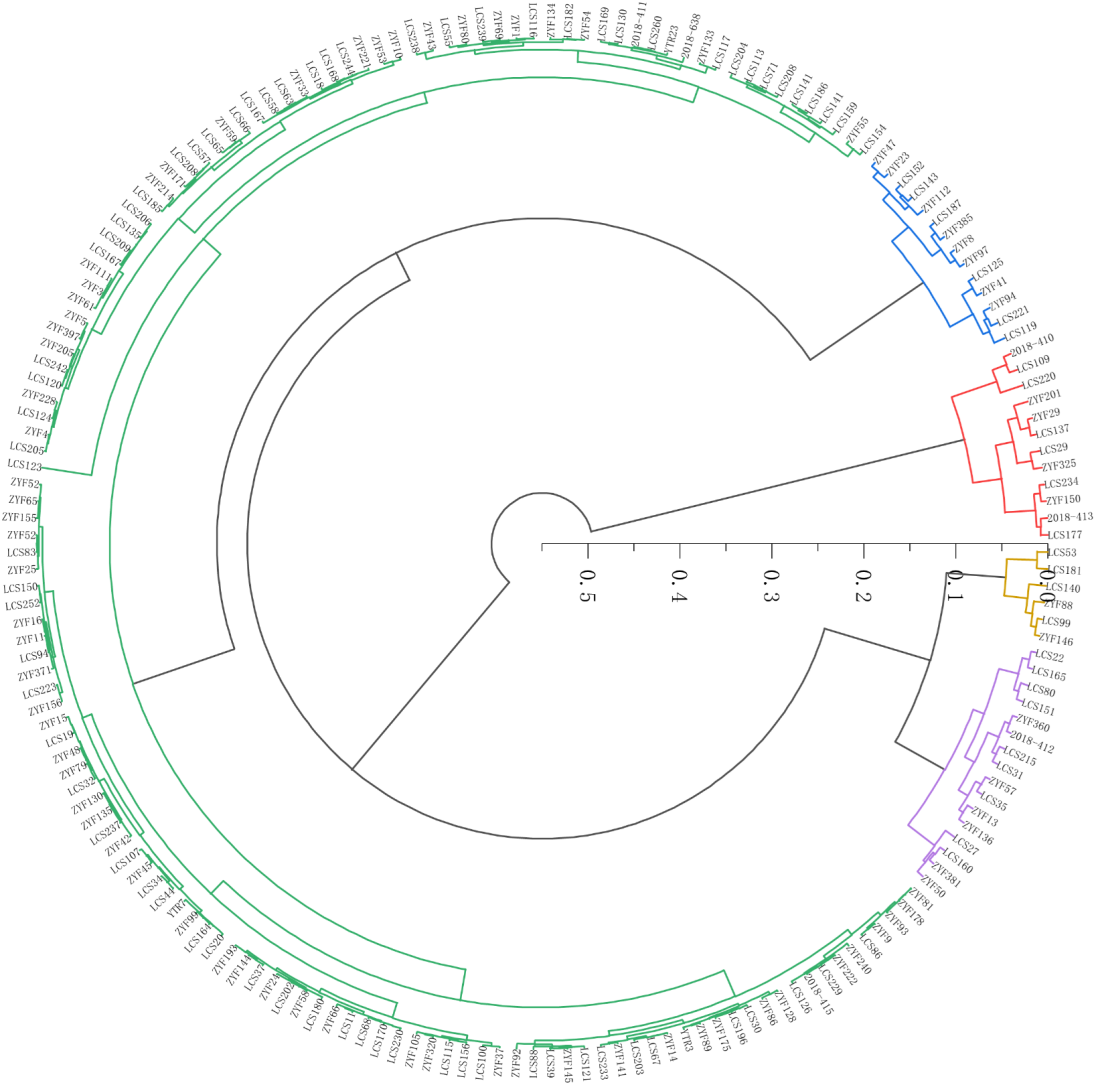
Cluster analysis map of salt tolerant ability of 188 sorghum germplasm. Red color indicates high salt tolerant (HT); blue color indicates salt tolerant (ST); green color indicates medium salt tolerant (MT); purple color indicates salt sensitive (SS) and yellow color indicates high salt sensitive (HS).

Based on the salt tolerant comprehensive evaluation value (D-value) of 188 sorghum germplasm accessions, cluster analysis was performed using Euclidean distance as the distance metric (**Figure 3**). The top-ranked sorghum germplasm had higher D-value, indicating that such sorghum germplasm had higher salt tolerance. In contrast, the lower D-value of the lower ranked sorghum germplasm indicated higher sensitivity to salt stress. Based on the grading method in Chen et al. (2012) study and using cluster analysis, 188 sorghum germplasm resources were categorized into five standard classes. Among them, 11 were highly salt-tolerant germplasm, 14 salt-tolerant germplasm, 142 moderately salt-tolerant germplasm, 38 salt-sensitive germplasm and 6 highly salt-sensitive germplasm (**Figure 3**). The highly salt-tolerant germplasms were LCS177, ZYF150, 2018-413, ZYF201, LCS234, LCS29, LCS137, ZYF29, LCS220, LCS325, and ZYF119; and the highly salt-sensitive germplasms were ZYF146, ZYF88, LCS99, LCS140, and LCS181, LCS53 (**Supplementary Table 4**).

### The osmotic substances differences in different salt tolerant varieties

**Figure 4:**
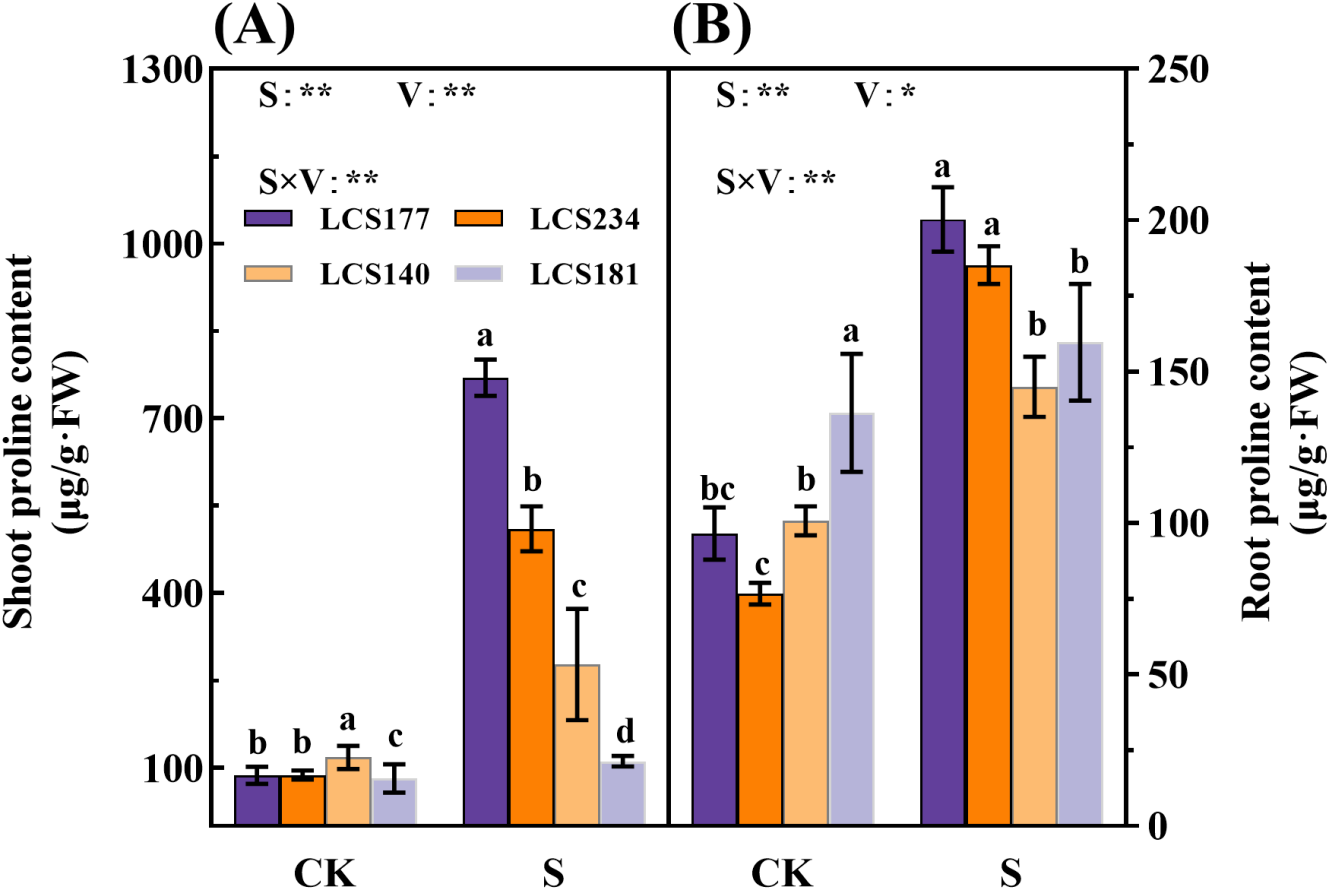
The proline content in stems **(A)** and roots **(B)** of different varieties under salt stress. CK represents normal culture level (0 mM NaCl), and S represents salt treatment (150 mM NaCl). Different lowercase letters indicate significant differences (P < 0.05), and the same lowercase letters indicate no significant differences (P < 0.05). ANOVAP values for salt treatment, cultivar, and their interaction are shown. *P<0.05; **P<0.01; ns, not significant.

**Figure 5:**
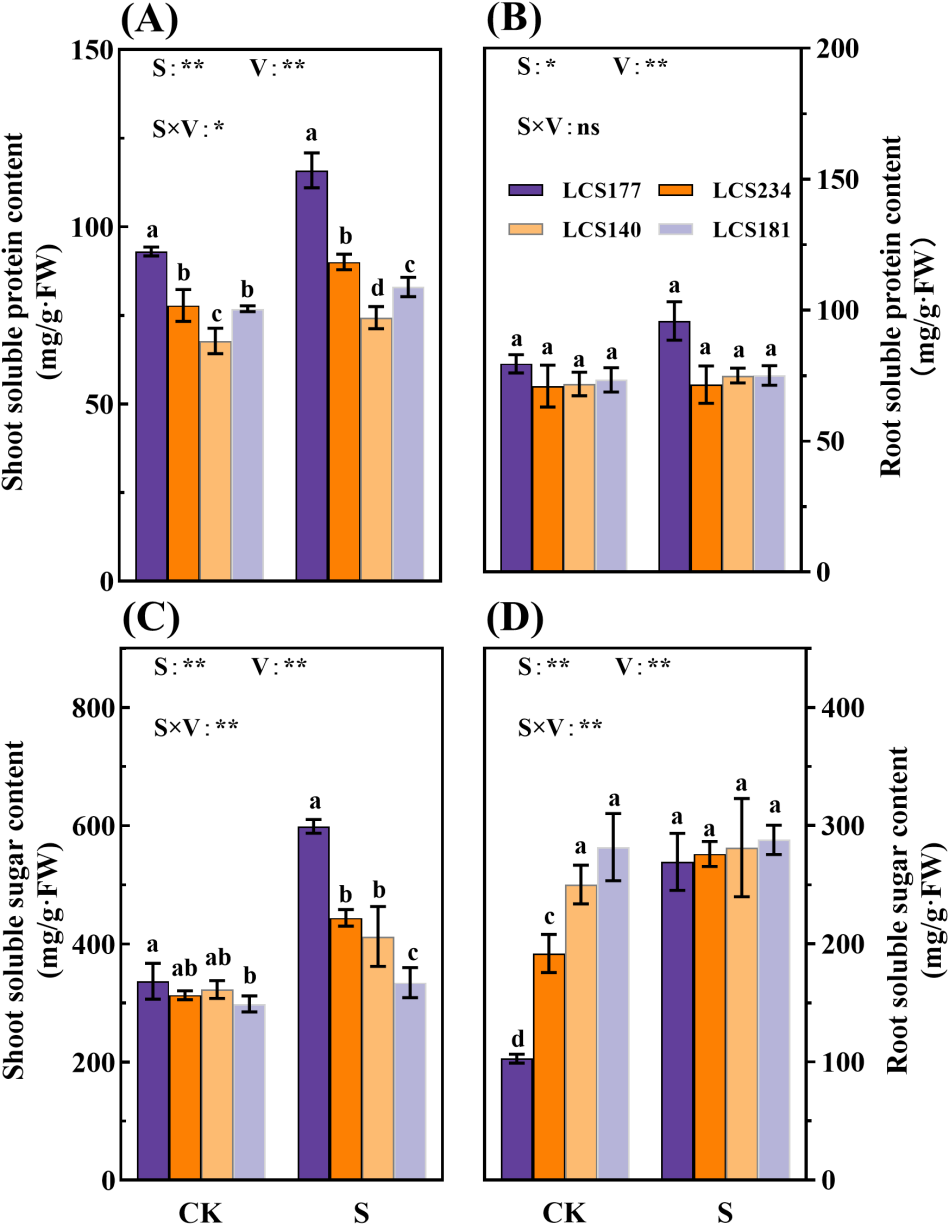
The soluble protein content **(A, B)** and soluble sugar content **(C, D)** in stems and roots of different sorghum varieties. CK represents normal culture level (0 mM NaCl), and S represents salt treatment (150 mM NaCl). Different lowercase letters indicate significant differences (P < 0.05), and the same lowercase letters indicate no significant differences (P < 0.05). ANOVAP values for salt treatment, cultivar, and their interaction are shown. *P<0.05; **P<0.01; ns, not significant.

Osmotic substances play an important role under salt stress and are key indicators for assessing sorghum salt tolerance. These substances maintain water balance by increasing cellular osmotic pressure and reducing the toxicity of sodium and chloride ions. Salt-tolerant varieties usually accumulate more osmotic substances, so by measuring the content of these substances, the salt tolerance of plants can be effectively judged. Under the salt treatment group (S), the shoot proline content of salt-tolerant varieties LCS177 and LCS234 increased to 784.95% and 483.1% compared to that of the control group (CK), respectively, whereas the proline content of the salt-sensitive varieties LCS140 and LCS181 only increased by 135.72% and 35.91%, respectively (**Figure 4A**). The salt-tolerant varieties LCS177 and LCS234 had higher proline content compared to the salt-sensitive varieties LCS140 and LCS181, indicating that the salt-tolerant varieties LCS177 and LCS234 responded faster to proline in response to osmotic stress (**Figures 4**, **5**). The trend of proline content in the roots was similar to that of the shoot (**Figure 4B**). Although proline content increased in all varieties under salt stress, salt-tolerant varieties LCS177 and LCS234 showed higher increases than salt-sensitive varieties LCS140 and LCS181 (**Figure 5**). In addition, the changes in soluble protein and soluble sugar followed a similar trend to that of proline.

As shown in **Figure 5**, the salt-tolerant varieties 177 and 234 exhibited a stronger salt stress response, with leaf soluble sugar content increasing by 77.8% and 41.8%, respectively, while the salt-sensitive varieties 140 and 181 showed only a 27.8% and 12.1% increase. Notably, the root soluble sugar content of salt-tolerant variety 177 increased by 162.2% after stress, which was significantly higher than the 2.2% increase observed in the salt-sensitive variety 181 (*P* < 0.05). Regarding soluble protein content, the leaf and root protein levels of salt-tolerant variety 177 increased by 24.6% and 20.6%, respectively, whereas those of salt-sensitive variety 181 increased by only 8.0% and 2.3%. These results indicate that salt-sensitive varieties exhibit insufficient accumulation of osmotic regulatory substances under stress conditions, particularly in root sugar accumulation and protein synthesis. Salt-tolerant varieties demonstrate a superior ability to maintain cellular osmotic homeostasis and coordinate stress responses.

### Comparative analysis of oxidative stress–related traits among cultivars under salt stress

**Figure 6:**
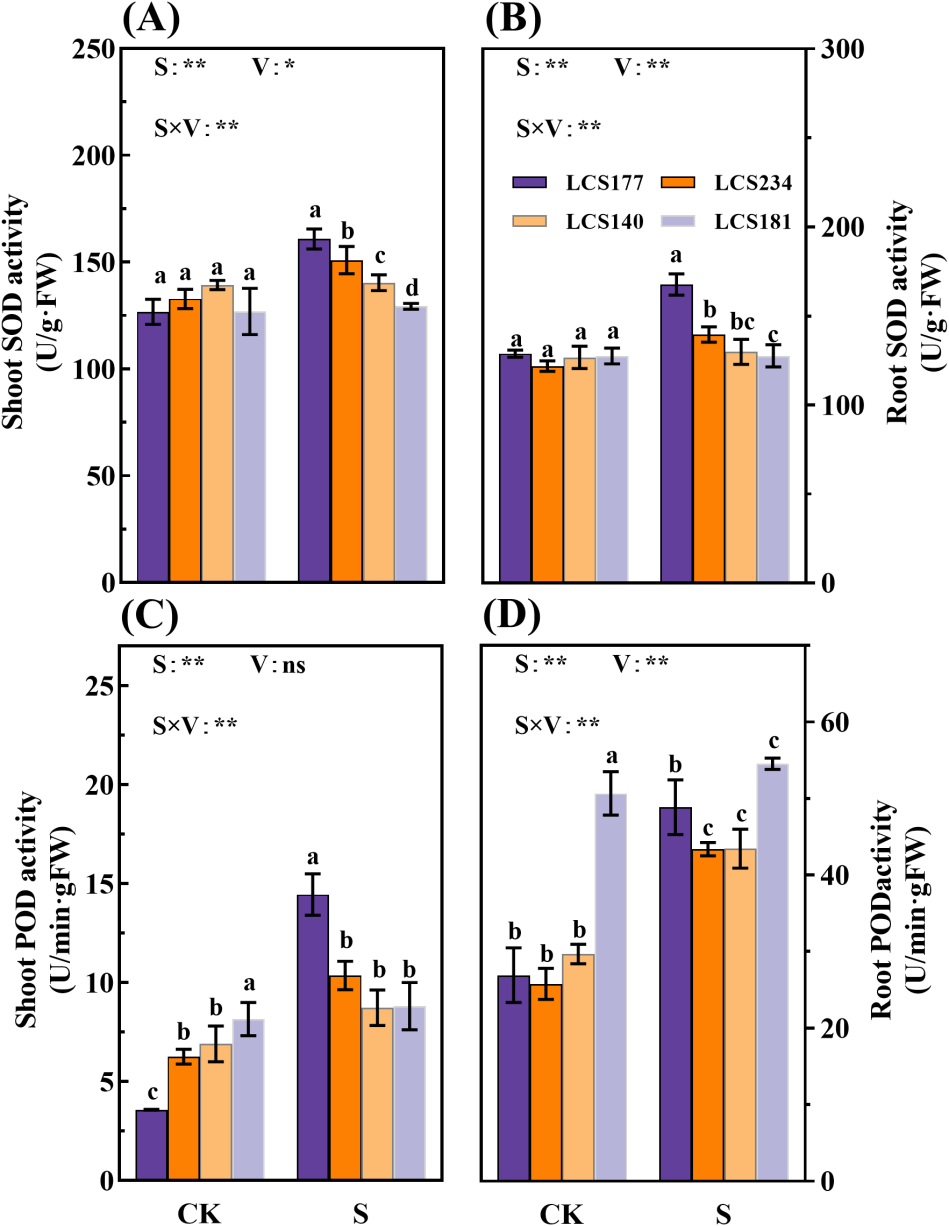
The SOD activity **(A, B)** and POD activity **(C, D)** in shoot and root of different sorghum varieties. CK represents normal culture level (0 mM NaCl) and S represents salt treatment (150 mM NaCl). Different lowercase letters indicate significant differences (P < 0.05), and the same lowercase letters indicate no significant differences (P < 0.05). ANOVAP values for salt treatment, cultivar, and their interaction are shown. *P<0.05; **P<0.01; ns, not significant.

Salt stress affected the activities of antioxidant enzymes as well as the levels of lipid peroxidation products (MDA) in salt-tolerant and salt-sensitive varieties. Under salt stress, sorghum seedlings accumulated large amounts of reactive oxygen species (ROS), leading to membrane peroxidation and activation of antioxidant enzyme activities. In this experiment, antioxidant enzyme activities were increased in sorghum seedlings of different varieties. The results showed that salt stress increased the activities of SOD by 26.94% and 13.67% in the shoot and 30.18% and 14.55% in the root of the salt-tolerant varieties LCS177 and LCS234, respectively (**Figures 6A, B**). However, the SOD activities in the shoot of salt-sensitive varieties LCS234 and LCS181 increased only by 0.77% and 1.88%, and the SOD activity in the roots increased only by 2.47% in LCS234, while there was no significant change in LCS181 (**Figures 6A, B**). Under normal culture conditions, there was no significant difference between shoot and root SOD activities of the four varieties (*P* > 0.05). However, under salt stress conditions, the SOD activity of salt-tolerant varieties was higher than of salt-sensitive varieties.

**Figure 7:**
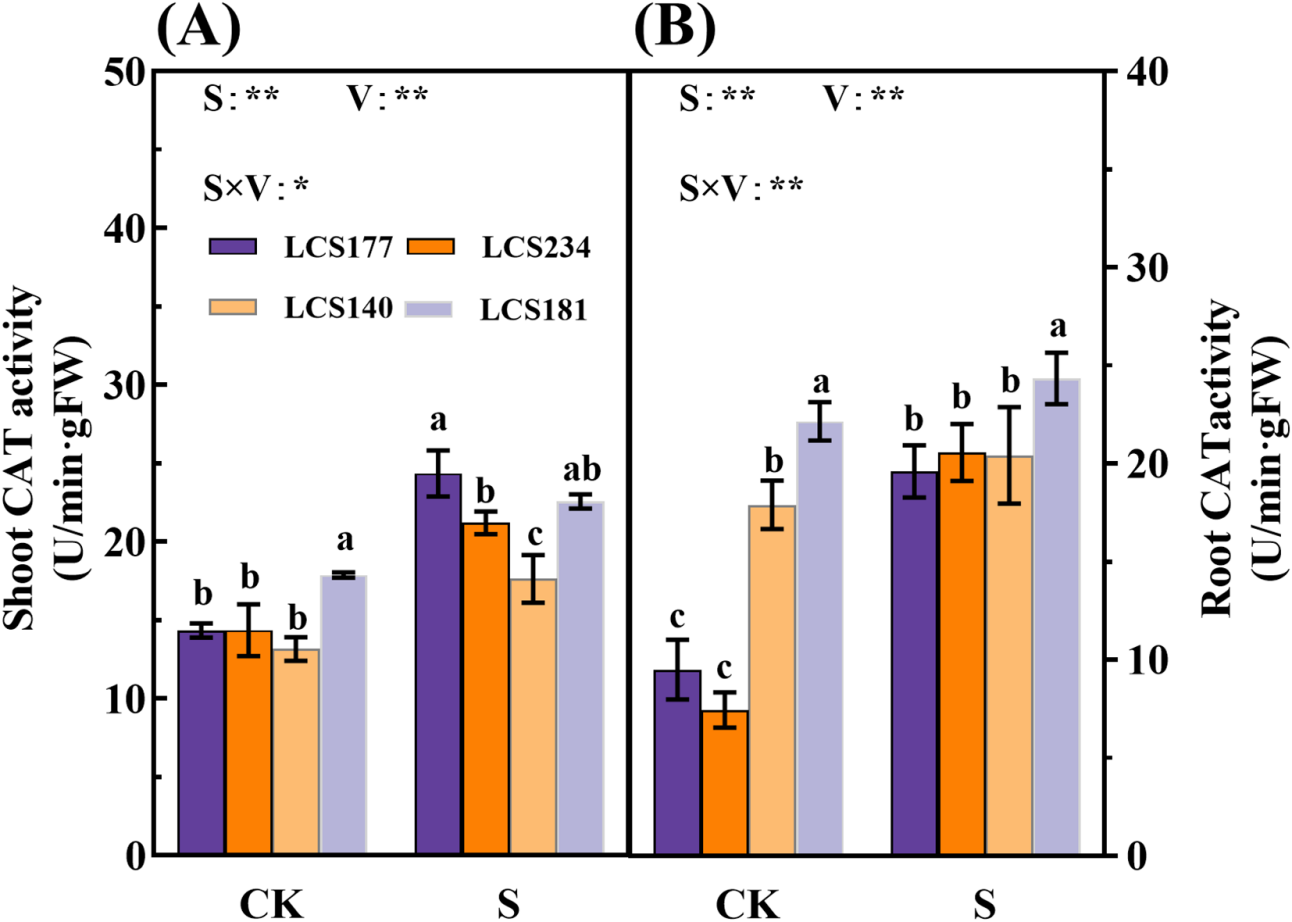
Changes in CAT activity **(A, B)** in stems and roots of different sorghum varieties. Different lowercase letters indicate significant differences (P < 0.05), and the same lowercase letters indicate no significant differences (P < 0.05). ANOVAP values for salt treatment, cultivar, and their interaction are shown. *P<0.05; **P<0.01; ns, not significant.

Meanwhile, salt stress significantly affects POD and CAT activities in shoot and root. Under salt stress, POD activities in shoot and root of two salt-tolerant varieties, LCS177 and LCS234, increased by 303.83% and 81.52%, 65.65% and 68.26%, respectively (**Figures 6C, D**). Meanwhile, salt-tolerant and salt-sensitive varieties showed similar trends of the salt stress respones in CAT activity (**Figures 7A, B**). Although the POD and CAT activities of shoot and root increased in all varieties under salt stress, the increases in LCS177 and LCS234 were higher than those in LCS140 and LCS181, which indicating that LCS177 and LCS234 had a greater capacity to cope with the accumulation of ROS under salt stress compared with LCS140 and LCS181.

**Figure 8:**
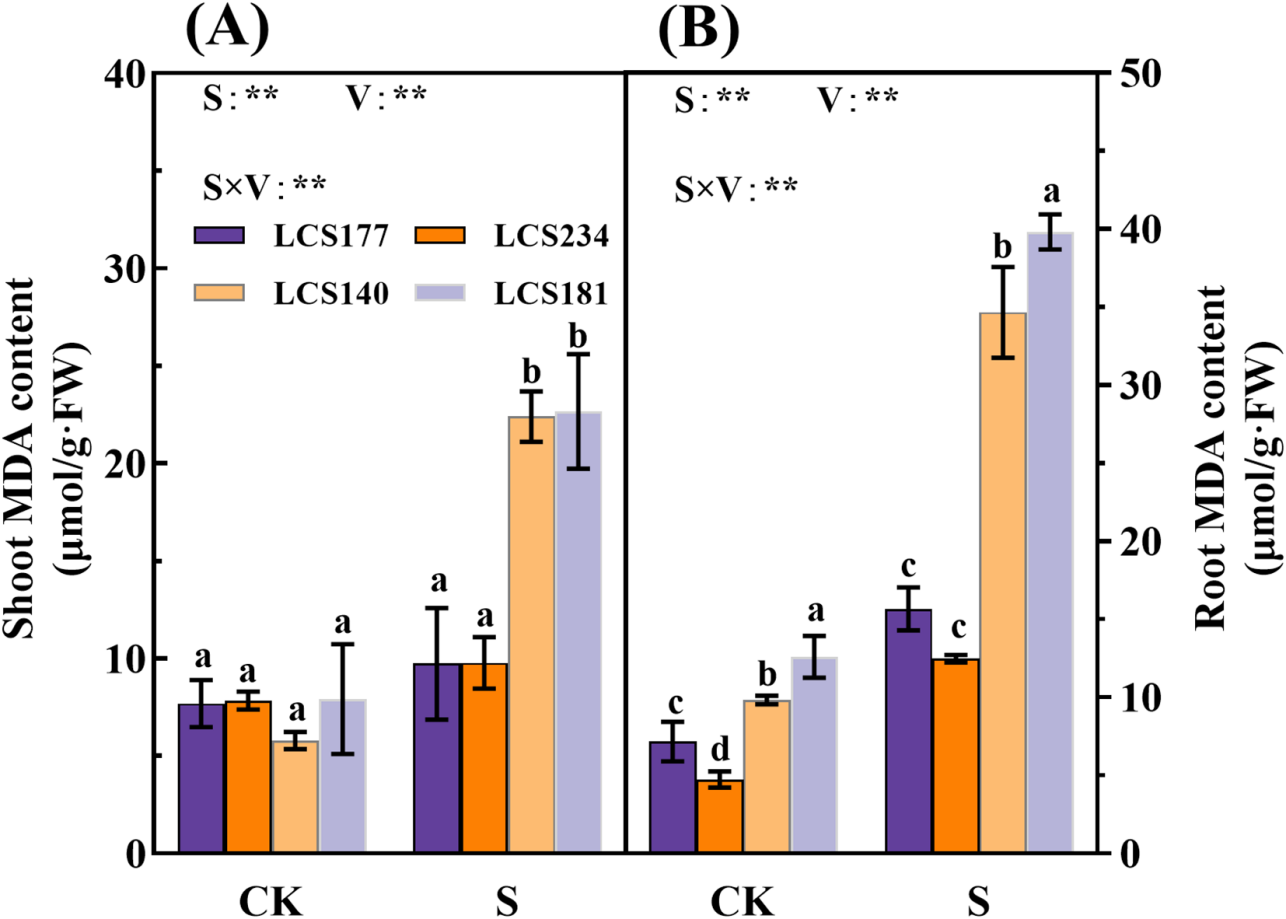
The MDA content **(A, B)** in shoot and root of different sorghum varieties. Different lowercase letters indicate significant differences (P < 0.05), and the same lowercase letters indicate no significant differences (P < 0.05). ANOVAP values for salt treatment, cultivar, and their interaction are shown. **P<0.01; ns, not significant.

Salt stress significantly affected the MDA content in the shoot and root of sorghum seedlings, and the average MDA activity of the two salt-sensitive varieties was higher than of the two salt-tolerant varieties. Compared to the control, the MDA content in the shoot and root of salt-sensitive varieties LCS140 and LCS181 increased by 287.9% and 252.91%, 186.28% and 216.69%, respectively. The increase in MDA content of salt tolerant varieties LCS177 and LCS234 was lower than of salt sensitive varieties LCS140 and LCS181.The increase in MDA content of salt tolerant varieties was not significant as compared to the control (*P* > 0.05) (**Figures 8A, B**).

## Discussion

### Evaluation of sorghum germplasm for salt tolerance

In recent years, sorghum has attracted a lot of attention due to its salt, alkali, drought, and barrenness tolerances (Stagnati et al., 2021). However, relatively few systematic studies have been conducted to characterize salt tolerance in sorghum germplasm. The main phenotype of crop response to adversity stress are changes in morphological and physiological characteristics, and there are significant differences in the selection of salt tolerance indicators among crops (Liu et al., 2020). In this study, we measured six morphological indicators—shoot length, root length, fresh weight of the shoot, fresh weight of the root, dry weight of the shoot, and dry weight of the root—of sorghum seedlings from 188 core sorghum germplasms under 150 mM NaCl stress treatment. The salt tolerance of these germplasms was comprehensively evaluated based on the changes in these morphological indicators under salt stress, using a combination of salt tolerance coefficients, correlation analysis, membership function values, principal component analysis, comprehensive evaluation, and cluster analysis. The salt tolerance of the germplasms was also graded. Finally, the salt tolerance of the selected germplasms was verified through the measurement of salt stress-related physiological indicators.

The inhibitory effect of salt stress on plant growth has been widely verified in a variety of crops (Rahman et al., 2021; Acosta-Motos et al., 2017; Hossain et al., 2018). Previous studies quantified the effects of salt stress on plants through toxic effects, osmotic effects, and phenotypic parameters (e.g., fresh weight, dry weight, root length, etc.), and established a standardized salt tolerance evaluation system based on the salt tolerance index, multi-indicator comprehensive evaluation, and cluster analysis. For example, in a salt tolerance study of 284 wheat lines, Choudhary et al. (2024) classified the germplasm into four salt tolerance classes through principal component analysis and clustering of 10 metrics such as germination rate and yield composition. Similarly, Wu et al. (2024) classified 40 celery germplasm into five salt-tolerant types using principal component and membership function analysis of 14 physiological and biochemical indicators. Kiani-Pouya et al., 2019, on the other hand, classified 114 quinoa germplasm at 3 levels by salt tolerance index (STI). Compared with the aforementioned crops, sorghum exhibits unique salt-tolerance advantages: in salt-tolerant types, efficient vacuolar accumulation of Na^+^ in the root elongation zone cells and delayed translocation to the shoot are observed, along with rapid generation of systemic signals that promote the accumulation of proline and antioxidant enzymes and enhance the translocation of sucrose from the shoot to the root system, thereby maintaining the coordination between photosynthesis and ion sequestration at the root tip (Abuslima et al., 2022). In contrast, salt-sensitive types fail to initiate timely vacuolar sequestration and lack rapid systemic signalling. Although different crops focus on distinct salt-tolerance indicators (e.g., wheat emphasizes yield traits, while tomato focuses on root biomass), the core lies in the consistency of multi-indicator dimensionality reduction, membership function standardization, and cluster grading.

This study employed a multidimensional analysis approach to systematically evaluate the salt tolerance of sorghum germplasm. Key phenotypic traits such as plant height and root length were selected to construct a comprehensive evaluation system. PCA was used to extract core factors with a cumulative contribution rate exceeding 80%, and the membership function method was applied to eliminate dimensional differences among indices. Based on cluster analysis, 188 sorghum germplasm accessions were classified into five salt tolerance levels: 11 highly salt-tolerant accessions, 14 salt-tolerant accessions, 142 moderately salt-tolerant accessions, 38 salt-sensitive accessions, and 6 highly salt-sensitive accessions. This classification is consistent with the three-level classification system used for quinoa and aligns with the methodologies established by Choudhary et al. (2024) in wheat research and Saeed et al. (2010) in tomato research, further confirming the reliability of the findings. Although the salt tolerance grading system based on multi-indicator comprehensive evaluation in this study is highly consistent with those of crops such as wheat and tomato in terms of methodology, the evaluation system established based on seedling data has certain limitations in predicting field performance. Salt tolerance mechanisms may vary across different growth stages, and salt tolerance at the seedling stage does not fully represent the salt tolerance of mature plants. For example, during the reproductive growth stage, the salt tolerance of sorghum may be influenced by physiological processes such as pollen development and seed filling, exhibiting different salt tolerance characteristics from those at the seedling stage. Therefore, the salt tolerance evaluation system in this study needs further validation during the reproductive and vegetative growth stages to ensure its applicability to mature plants. Additionally, the complexity of field environments (such as soil type, water status, and temperature changes) may also affect the salt tolerance of sorghum. These factors need to be comprehensively considered in field experiments to further refine the salt tolerance evaluation system.

In summary, the salt tolerance classification system based on comprehensive evaluation of multiple indicators has cross-crop generalizability. The classification method in this study is consistent with previous studies in terms of the core steps, and although the selection of indicators was slightly adjusted due to crop characteristics, the strict alignment of the methodological logic guarantees the comparability and accuracy of the results and provides a screening criterion that can be horizontally referenced for sorghum salt tolerance breeding.

### Physiological response of sorghum germplasm to salt stress

Under external environmental stress, plants often respond to unfavorable conditions by regulating their physiological and metabolic pathways. This adaptive response is an important survival strategy for plants in the face of adversity (Singh et al., 2020). Plants respond to a variety of environmental stresses through osmoregulatory mechanisms involving the dynamic balance of water and solutes inside and outside the cell and the regulation of cell membrane permeability, thereby enhancing their adaptability to saline and alkaline stress (Li et al., 2022). For example, Osmotic regulating substances regulate osmotic pressure by preventing dehydration of organelles and stabilizing proteins and cell membranes, thereby improving plant survival under extreme stress conditions (Singh et al., 2015). In this study, salt stress significantly promoted the accumulation of osmotic regulating substances such as proline, soluble proteins and soluble sugars compared to the control. The highly salt-tolerant varieties LCS177 and LCS234 accumulated significantly more than the highly salt-sensitive varieties LCS140 and LCS181 (**Figures 5**, **6**). These results suggest that the more salt-tolerant the crop, the greater the accumulation of osmotic substances. Similar accumulation trends of soluble proteins and soluble sugars content under salt stress were also reported (Abdel Latef et al., 2016).

Scavenging ROS is essential for plant defense against oxidative damage under stressful conditions. Extreme environments lead to significant accumulation of ROS in plants, which in turn damage intracellular macromolecules (Kavian et al., 2022). However, plants are able to protect cells from further oxidative damage through their enzymatic defense system (Wang et al., 2022). In this study, we found that MDA content of sorghum shoots and root increased significantly under salt stress, especially in varieties LCS140 and LCS181. This indicates that salt-sensitive varieties are prone to accumulate large amounts of ROS under salt stress, leading to lipid peroxidation injury to generate MDA (Kumar et al., 2023). At the same time, the highly salt-tolerant varieties LCS177 and LCS234 exhibited significant elevated SOD, POD, and CAT activities under salt stress, whereas the highly salt-sensitive varieties LCS140 and LCS181 showed a smaller increase in antioxidant enzyme activities. The results indicated that salt-tolerant varieties rapidly scavenged ROS and mitigated oxidative damage through enhanced antioxidant enzyme activities. In contrast, salt-sensitive varieties suffered more severe damage under salt stress due to their lower antioxidant enzyme activities.

Although salt-tolerant lines exhibit higher proline and antioxidant enzyme activities under salt stress, such responses may incur potential growth costs. Under non-stress conditions, the growth rates of salt-tolerant varieties LCS177 and LCS234 may be slightly lower than those of salt-sensitive varieties LCS140 and LCS181. This growth cost may arise from the higher energy and resource consumption required by salt-tolerant varieties to maintain the accumulation of osmoregulatory substances and the activity of antioxidant enzyme systems (Cai and Gao, 2020). Additionally, the mechanism of ion exclusion may be one of the unique salt tolerance mechanisms in sorghum. Sorghum can expel excess sodium ions from cells through ion channels on the cell membrane, thereby reducing the intracellular salt concentration. This ion exclusion mechanism is not commonly found in other crops and may be an important physiological basis for sorghum’s salt tolerance. Future research can further explore the molecular mechanisms and regulatory pathways of this ion exclusion mechanism, providing new theoretical foundations for salt-tolerant breeding in sorghum (Calone et al., 2020; Peduzzi et al., 2024).

## Conclusion

In this study, we assessed the salt tolerance of sorghum seedlings by treating them with 150 mM NaCl and constructed a salt tolerance evaluation system. Salt tolerance of 188 sorghum varieties was analyzed by combining SI, Spearman’s correlation analysis, membership function analysis, PCA, comprehensive evaluation, and cluster analysis methods. The results showed that the salt tolerance ranking of sorghum varieties was determined by PCA and comprehensive evaluation, and the varieties were categorized into five salt tolerance classes using cluster analysis: highly salt tolerant (e.g., LCS177, LCS234), salt tolerant, moderately salt tolerant, salt sensitive, and highly salt sensitive (e.g., LCS140, LCS181). Although all varieties showed an increase in osmotic substances and antioxidant enzyme activities under salt stress, the salt-tolerant varieties showed significantly higher relevant indexes than the salt-sensitive varieties, and the latter had significantly higher MDA levels. The stronger physiological and metabolic responses exhibited by the salt-tolerant varieties may be the key factor for their higher salt tolerance, revealing the potential basis of their salt tolerance mechanism.

## Data Availability

The original contributions presented in the study are included in the article/**Supplementary Material**. Further inquiries can be directed to the corresponding authors.

## Supplementary material

Supplementary Table 1 Effect of NaCl treatment on germination parameters of 188 sorghum germplasms.

Supplementary Table 2 Salt tolerance coefficients of 188 sorghum accessions.

Supplementary Table 3 Factor scoring system matrix.

Supplementary Table 4 Comprehensive index value, membership function value, D value and evaluation o fdifferent Sorghum varieties.
